# The extended functions of *CYCLOIDEA*-like genes in the control of floral architecture

**DOI:** 10.1093/plcell/koad142

**Published:** 2023-05-18

**Authors:** Humberto Herrera-Ubaldo

**Affiliations:** Assistant Features Editor, The Plant Cell, American Society of Plant Biologists, Rockville, MD, USA; Department of Plant Sciences, University of Cambridge, Cambridge CB2 3EA, UK

Flowers can have many colors, shapes, and features, but flower symmetry is a critical feature associated with pollinator specialization and specification. In nature, there are radially symmetric flowers (actinomorphic flowers, i.e. roses), with several symmetry planes, and bilateral symmetric flowers (zygomorphic flowers, i.e. *Antirrhinum*), with only 1 symmetry plane (Endress 2001). At the molecular level, the TCP transcription factor family members *CYCLOIDEA* (*CYC*) and *DICHOTOMA* (*DICH*) are central players in the regulatory module controlling bilateral symmetry (Luo et al. 1996, 1999). However, whether they establish other flower features, such as floral orientation and color patterns, is still unknown.

In this issue, **Xia Yang, Yang Wang, Tian-Xia Liu, and colleagues** (Yang et al. 2023) used zygomorphic *Chirita pumila* flowers to explore how *CYC*-like genes control symmetry, orientation, pigmentation, and nectar guide formation. The flowers in *C. pumila* are zygomorphic, with contrasting petal and stamen features. The dorsal and ventral petals are shorter, and the dorsal petals are wider. The development of dorsal and lateral stamens is delayed, and only 2 ventral stamens generate fertile anthers. *C. pumila* flowers orient horizontally, and like many other zygomorphic flowers, color patterns are asymmetrical. Additionally, a ridge is formed in the dorsal corolla, and a bulge in the dorsal petal and the lamella generates a groove that holds the style.

The authors first focused on the expression patterns of 2 *CYC*-like TCP genes, *CpCYC1* and *CpCYC2* (orthologs of *Antirrhinum majus CYC*/*DICH* genes), using RNA in situ hybridization. During flower development, the expression of *CpCYC1* was first detected in the floral meristem, then in the sepal primordia, later in the petal and stamen primordia, and finally was restricted to the adaxial epidermis of dorsal petals as well as the dorsal and lateral stamens. The expression of *CpCYC2* was similar but weaker.

Next, *CpCYC1* and *CpCYC2* were ectopically expressed to assess their functional roles. The overexpression (OE) of *CpCYC1* caused flower dorsalization at varying degrees; the flowers had wider petals, lacked the typical yellow coloration, and had increased lamella number and infertile stamens; additionally, the floral orientation shifted more upward. Similar phenotypes were observed in lines overexpressing *CpCYC2*, except in petal color; *CpCYC1* OE lines produced purple petals, whereas *CpCYC2* OE lines had pale ones. Interestingly, both OE lines displayed high levels of *CpCYC1*, indicating the upregulation of *CpCYC1* by both CpCYC1 and CpCYC2. These results suggest that the dorsalization is related to the ectopic expression of both *CpCYC1* and *CpCYC2*.

Contrastingly, the downregulation of *CpCYC1* or *CpCYC2* using fusions with the SRDX repression domain generated ventralized actinomorphic flowers: all petals acquired ventral identity, the yellow spots were extended, and all the stamens were fertile; however, the flower orientation did not change. A *cyc1 cyc2* double mutant constructed by gene editing displayed fully ventralized flowers with upward orientation and uniform yellow pigmentation. Together with the phenotypes of *cyc1* and *cyc2* single mutants, these results indicated *CpCYC1* has a major role in controlling flower symmetry, orientation, and nectar guide patterns.

The pigment distribution in the petals and the changes to petal pigmentation in the *CpCYC1/2* OE lines suggested the *CpCYC1/2* genes also control coloration. The authors performed RNA-seq analyses in wild-type lines to identify the biochemical pathway acting downstream of *CpCYC1/2* genes. Biochemical analyses indicated flavonoids are responsible for the yellow pigmentation in the corolla tube. Five flavonoid synthesis-related genes were highly expressed in the yellow nectar guides of the flowers. A *flavonoid 3′,5′-hydroxylase* (*CpF3′5′H*) gene was upregulated in the *cyc1 cyc2* double mutant and undetectable in the *CpCYC1/2* OE lines, suggesting a direct CpCYC1/2 gene target. Electrophoresis mobility shift assays confirmed CpCYC1 and CpCYC2 binding to the promoter of *CpF3′5′H*. Luciferase reporter assays demonstrated that CpCYC1 or CpCYC2 binding leads to repressed *CpF3′5′H* expression.

**Figure 1.**
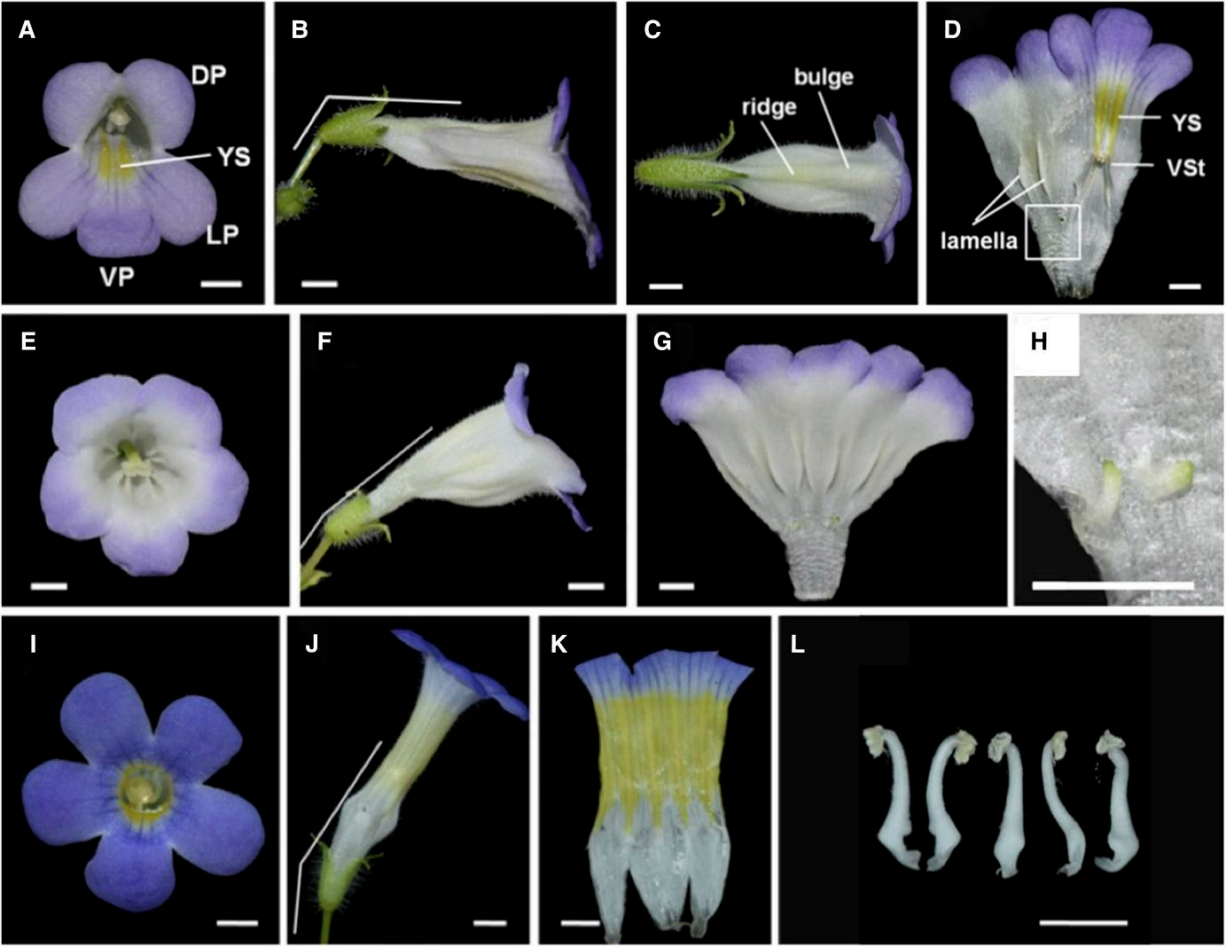
**A–D**) Wild-type flower of *C. pumila*. **E–H**) Flower of the *d35Spro:CpCYC1* line with dorsalized actinomorphic flowers. **I–L**) Flower of the *cyc1 cyc2* double mutant with ventralized actinomorphic flowers. DP, dorsal petal; LP, lateral petal; VP, ventral petal; VSt, ventral stamen.; YS, yellow spot. Bars represent 0.5 cm. Adapted from Yang et al. (2023), Figures 1, 3, 5.

To test CYC functional conservation, the authors used *Primulina heterotricha*, which has flowers similar to *C. pumila* in the dorsal ridge, the bulge, ventral coloration, and flower orientation. The overexpression of *PhCYC1C* and *PhCYC1D* in *C. pumila* produced dorsalized flowers lacking yellow pigmentation with an upward direction, resembling the *CpCYC1* overexpression. These results demonstrate the conserved role of *CYC-like* genes in controlling multiple aspects of zygomorphic flower development (Fig. 1).
